# G2-S16 Polyanionic Carbosilane Dendrimer Can Reduce HIV-1 Reservoir Formation by Inhibiting Macrophage Cell to Cell Transmission

**DOI:** 10.3390/ijms22168366

**Published:** 2021-08-04

**Authors:** Ignacio Relaño-Rodríguez, María de la Sierra Espinar-Buitrago, Vanessa Martín-Cañadilla, Rafael Gómez-Ramírez, María Ángeles Muñoz-Fernández

**Affiliations:** 1 Section Head Immunology, Laboratorio InmunoBiología Molecular, Hospital General Universitario Gregorio Marañón (HGUGM), 28007 Madrid, Spain; ignaxete@hotmail.com (I.R.-R.); marisierri90@gmail.com (M.d.l.S.E.-B.); vcmartin.93@gmail.com (V.M.-C.); 2 Instituto de Investigación Sanitaria Gregorio Marañón (IiSGM), 28007 Madrid, Spain; 3 Departamento de Química Orgánica y Química Inorgánica, Instituto de Investigación Química “Andrés M. del Río” (IQAR), Universidad de Alcalá (UAH), 28871 Alcalá de Henares, Spain; rafael.gomez@uah.es; 4 Networking Research Center on Bioengineering, Biomaterials and Nanomedicine (CIBER-BBN), 28029 Madrid, Spain; 5 Spanish HIV-HGM BioBank, Hospital General Universitario Gregorio Marañón C/Dr. Esquerdo 46, 28007 Madrid, Spain

**Keywords:** macrophages, HIV reservoir, nanotechnology, dendrimers

## Abstract

Human immunodeficiency virus (HIV-1) is still a major problem, not only in developing countries but is also re-emerging in several developed countries, thus the development of new compounds able to inhibit the virus, either for prophylaxis or treatment, is still needed. Nanotechnology has provided the science community with several new tools for biomedical applications. G2-S16 is a polyanionic carbosilane dendrimer capable of inhibiting HIV-1 in vitro and in vivo by interacting directly with viral particles. One of the main barriers for HIV-1 eradication is the reservoirs created in primoinfection. These reservoirs, mainly in T cells, are untargetable by actual drugs or immune system. Thus, one approach is inhibiting HIV-1 from reaching these reservoir cells. In this context, macrophages play a main role as they can deliver viral particles to T cells establishing reservoirs. We showed that G2-S16 dendrimer is capable of inhibiting the infection from infected macrophages to healthy T CD4/CD8 lymphocytes by eliminating HIV-1 infectivity inside macrophages, so they are not able to carry infectious particles to other body locations, thus preventing the reservoirs from forming.

## 1. Introduction

HIV-1 has become one of the main pandemics in modern times. The Joint United Nations Program on HIV/AIDS (UNAIDS) concluded that there are 1.7 million new infections and 37.9 million people living with HIV-1 infection [1]. After the implementation of combination antiretroviral therapy (ART), the pathological outcome of HIV-1 infection has improved substantially; however, a functional cure for HIV-1 has not been achieved. For this reason, finding a more accessible and long-lasting prevention/therapeutic approach against HIV-1 infection is one of the biggest current challenges of the biomedical community.

In this sense, multiples approaches are being studied to fight against HIV-1, from the elimination of the pathogen during different stages of infection, to prophylaxis, as well as eliminating the focus of latency. Including all this, nanotechnology has the capability to provide great advances and in particular, the use of dendrimers should be highlighted [2,3,4]. Dendrimers are actively developing, with promising anti-HIV-1 activity demonstrated in vitro. In particular, carbosilane dendrimers have shown great efficacy as microbicides, specifically G2-S16 [5]. This second-generation dendrimer with a silica core and 16 sulfonate end groups has demonstrated in previous assays that it is capable of destabilizing the GP120-CD4 complex by blocking HIV-1 entry and cell-to-cell fusion [6,7,8] (reviewed in [9]).

Based on the mechanism of action of this dendrimer against HIV-1 both in vitro and in vivo, we studied the possibility that our G2-S16 dendrimer was able to inhibit the HIV-1 within macrophages. Macrophages play a major role as potential viral reservoirs, not only because they appear to have greater resistance to cytopathic effects than T cells [10,11], but they also are capable of containing competent viruses for weeks in lymphoid organs, preventing the interaction of ART. Thus, during the acute phase of HIV-1 infection, macrophages establish primary infection but perivascular macrophages deliver the virus to different organs, including the brain [12,13], establishing itself as an immune sanctuary [14].

They also undergo viral accumulation in cellular compartments connected to the surface, inaccessible to neutralizing antibodies [15]. It should be noted that the virus is internalized in exosomes or microvesicles to facilitate and improve spread, establishing itself as mechanisms of immune evasion. Therefore, HIV-1-infected macrophages can cause viral rebound after discontinuation of ART.

In addition to acting as HIV-1 viral reservoirs, infected macrophages can transmit HIV-1 to T CD4/CD8 cells through cell-to-cell contact, leading to a 10-fold higher rate of infection than free cell virus [16,17,18]. Although activated T CD4/CD8 cells are the main target cells of the virus, both the location and function of macrophages make it possible to generate a continuous infection of T cells through continuous cell–cell interactions [19,20]. In this way, we investigated if G2-S16 dendrimer could prevent the spread of HIV-1 when macrophages perform their function as an antigen-presenting cell for CD4 / CD8 T lymphocytes.

## 2. Results and Discussion

### 2.1. Biocompatibility of G2-S16 Dendrimer

Previous published work has demonstrated the biocompatibility of G2-S16 dendrimer in several cell lines, as well as human primary cell cultures. The maximum non-toxic concentrations for almost all cell lines (HEC-1A, HeLa, VK2/E6E7, Ect1/E6/E7, End1/E6E7, and TZM.bl) and primary human cells (PBMCs, CD4+ T lymphocytes, Treg, monocytes, MDMs, and MDDCs) were up to 20 µM [21,22,23]. However, as described in our previous work, we chose 10 µM as this was the maximum inhibitory concentration obtained in several studies in vitro [5,6,7].

### 2.2. G2-S16 Dendrimer Internalizes into Monocyte-Derived Macrophages and Is Stable for 48 h

**Figure 1:**
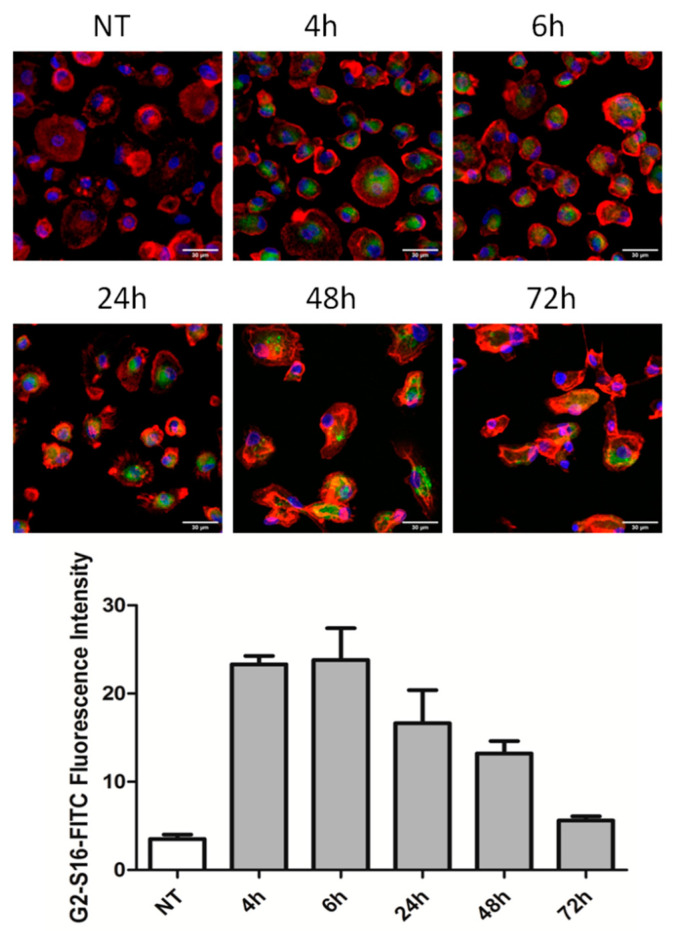
Representative confocal images of G2-S16 internalization in MDMs. Human monocytes were isolated and differentiated to MDMs. After differentiation, MDMs were treated with G2-S16-FITC for 2 h and washed with glycine (pH 3) to detach all non-internalized dendrimer in the cell membrane and washed 3 times with PBS. Confocal images were taken at 4, 6, 24, 48, and 72 h after treatment. FITC fluorescence was measured and is represented in the bars. Red: cell membrane. Blue: cell nuclei. Green: G2-S16-FITC dendrimer. Representative images of 3 independent experiments are shown.

In order to study the internalization of G2-S16 dendrimer into MDMs, fluorescence images of confocal microscopy were taken (Figure 1). G2-S16-FITC dendrimer was incubated with MDMs for 2 h, washed with glycine (pH 3) to eliminate the non- internalized G2-S16 dendrimer, and analyzed by confocal microscopy at different times post incubation. Results showed that G2-S16 dendrimer is able to internalize into MDMs at relatively short times and it remains inside the cell for at least 72 h. We observed high fluorescence intensity for the first 4, 6, and 24 h, indicating high absorption of G2-S16 dendrimer. At 48 h post-treatment, the fluorescence intensity began to diminish, but we still observed a high quantity of G2-S16 dendrimer inside the cells. As reported in the scientific literature, viral particles are able to resist inside macrophages, maintaining the ability to infect new T lymphocytes [24,25,26]. In our results, we observed at 72 h post-treatment that fluorescence intensity decays to low levels but is still observable using confocal microscopy. However, the G2-S16-FITC molecule stability in G2-S16 dendrimer is not as high as its sulfonate groups, thus meaning that this reduced fluorescence may be caused by loss of the G2-S16-FITC molecule but not the G2-S16 dendrimer structure or functional groups. This result indicates that G2-S16 dendrimer is capable of internalizing into MDMs, being stable for at least 48 h. Thus, this indicates that in the main window of HIV-1 spreading from macrophages to other tissues or circulating cells, our G2-S16 dendrimer is still inside the cell and probably maintaining its antiviral activity.

### 2.3. G2-S16 Dendrimer Internalized into Monocyte-Derived Macrophages Eliminates HIV-1 Infectivity

**Figure 2:**
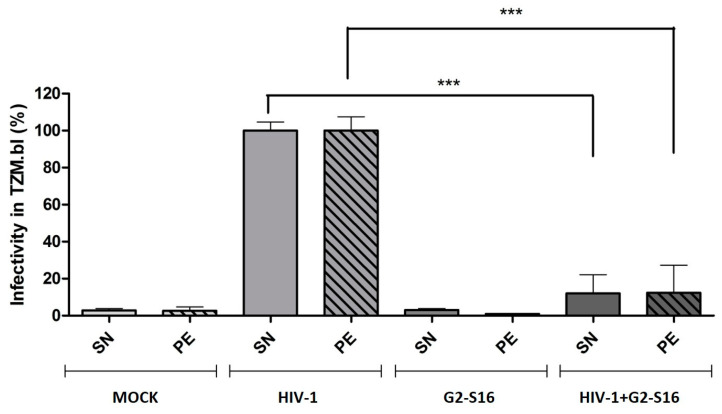
G2-S16 inhibits HIV-1 inside MDMs. Human monocytes were isolated and differentiated to MDMs, after differentiation cells were treated for 2 h with G2-S16 dendrimer, washed with glycine (pH 3) to detach non-internalized dendrimer, and washed with PBS. MDMs were then infected with 6 ng/µL of HIV-1 per 1 × 10^6^ cells for 2 h, inoculum was discarded, and cells incubated at 37 °C for 3 days. Supernatants and cell pellets were separated by centrifugation and titrated by TZM.bl infection. Mock cells were used as non-treated control. The mean values (mean ± SD) of three independent experiments are shown (*** *p* < 0.001).

To study if G2-S16 dendrimer maintains its ability to inhibit HIV-1 internalized by MDMs, we analyzed the remaining virus in supernatants (SN) and cell pellets (PE) obtained on inhibition assays by titration on TZM-bl cells. Briefly, TZM.bl cells were seeded, incubated for 24 h, and treated with 100 µL of supernatants or cell pellets for 48 h. Viral infectivity on TZM.bl was measured by luciferase assay. Our result (Figure 2) showed that G2-S16 dendrimer is capable of inhibiting almost 85–90% of the internalized HIV-1 viral particles. We observed a reduction of more than 80% in supernatants, meaning that a very reduced number of viral particles are released to extracellular medium with G2-S16 dendrimer treatment. In addition, the results obtained when cell pellets were analyzed showed the same reduction, reaching almost 85%. Thus, this means that G2-S16 dendrimer is not only able to inhibit HIV-1 release to extracellular media but is also capable of practically eliminating the infectivity of the remaining viral particles inside MDMs.

### 2.4. Infected Monocyte-Derived Macrophages Do Not Spread HIV-1 Infection to T CD4/CD8 Lymphocytes after G2-S16 Dendrimer Treatment

**Figure 3:**
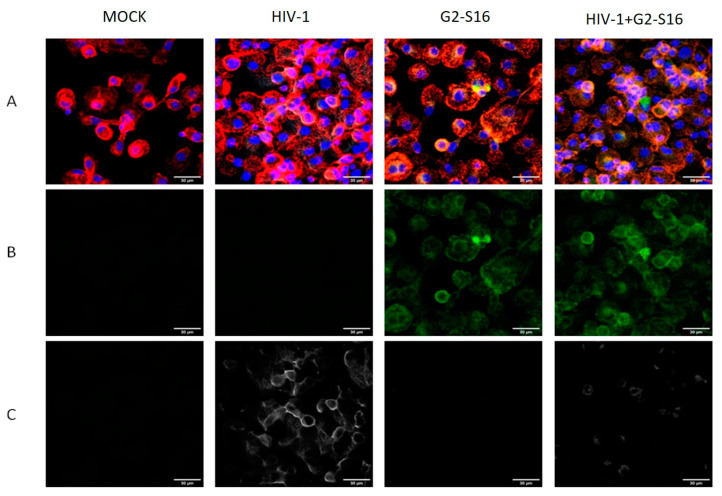
Representative confocal images of HIV-1 infection in MDMs and T cell co-cultures. Human monocytes and T lymphocytes were isolated from the same buffy coat. Monocytes were differentiated to MDMs. After differentiation, MDMs were treated with G2-S16-FITC for 2 h and washed with glycine (pH 3) to detach all non-internalized dendrimer in the cell membrane and washed 3 times with PBS. After washes, MDMs were infected with 6 ng/µL per 1 × 10^6^ cells of HIV-1 and co-cultured with T lymphocytes for 48 h. Supernatant was gently discarded and cells were mounted to perform confocal microscopy. Confocal images were taken 48 h after co-culture with T cells. (**A**) Merge (**B**) G2-S16-FITC (**C**) HIV-1 P24. Red: cell membrane. Blue: cell nuclei. Green: G2-S16/G2-S16-FITC dendrimer. White: HIV-1 p24 protein Scale bar: 30µm. Representative images of 3 independent experiments are shown.

Infected macrophages are one of the main factors in HIV-1 spreading to different tissues and organs [27]. This is due to macrophages’ ability to carry the virus without losing infectivity for long times, leading to a higher infectivity when they contact with CD4/CD8 T lymphocytes due to the cellular synapses [28,29]. As commented before, these synapses protect viruses from antiviral molecules and immune defenses present in the extracellular medium and are not capable of internalizing into the cell [15]. To study if G2-S16 dendrimer is able to inhibit cell-to-cell infection of T lymphocytes from MDMs, we performed co-cultures of MDMs infected and G2-S16 dendrimer treated with T lymphocytes obtained from the same buffy coat. We first performed confocal images to determine if HIV-1 particles were able to be released from MDMs and reach CD4/CD8 T cells. In our results (Figure 3), we found that MDMs treated with G2-S16 dendrimer (green) and HIV-1 infected present very low viral particles (white), in contrast with HIV-1-infected MDMs, which present high fluorescence intensity, meaning that they preserve viral particles in the internal membrane. We observed that most remaining viral particles co-localize with G2-S16 dendrimer, thus confirming our previously published work describing that G2-S16 dendrimer is able to form complex with viral gp120 [7,30].

**Figure 4:**
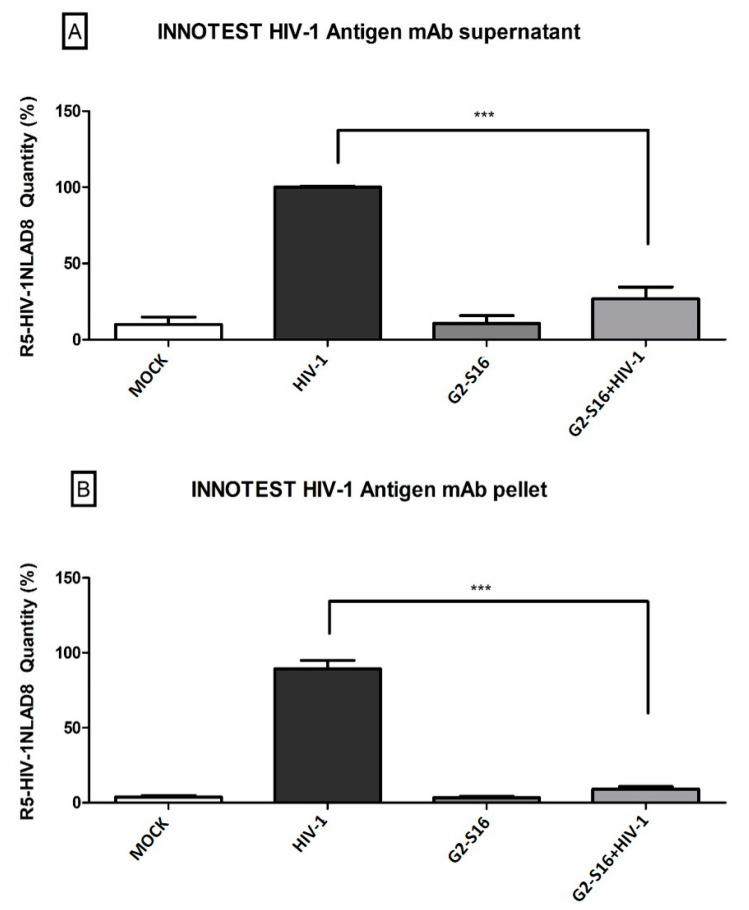
G2-S16 decreases the HIV-1 amount reaching T cells from infected MDMs. MDMs treated with G2-S16 and infected with 6 ng/µL of HIV-1 per 1 × 10^6^ cells for 2 h were co-cultured with isolated T CD4/CD8 for 48 h. After incubation with T cells, (**A**) supernatant and (**B**) pellets were isolated and titrated by HIV-1 p24 enzyme immunoassay (Elisa kit INNOTEST HIV-1 Antigen mAb). Mock cells were used as the non-treated control. The mean values (mean ± SD) of three independent experiments are shown (*** *p* < 0.001).

To confirm that the viral particles could not reach CD4/CD8 T cells, lymphocytes were isolated from MDMs by several washes, verified by microscopy that T cells were isolated correctly, and analyzed by two different techniques. First, Elisa enzyme immunoassay was performed to determine the amount of p24 protein present in TCD4/CD8 lymphocytes. Our results show that treatment with G2-S16 reduced the amount of p24 protein, both for supernatants and pellets. In fact, p24 protein values in the pellets did not exceed 10%, which shows that G2-S16 dendrimer significantly reduces the quantity of virus that reaches T cells (Figure 4).

**Figure 5:**
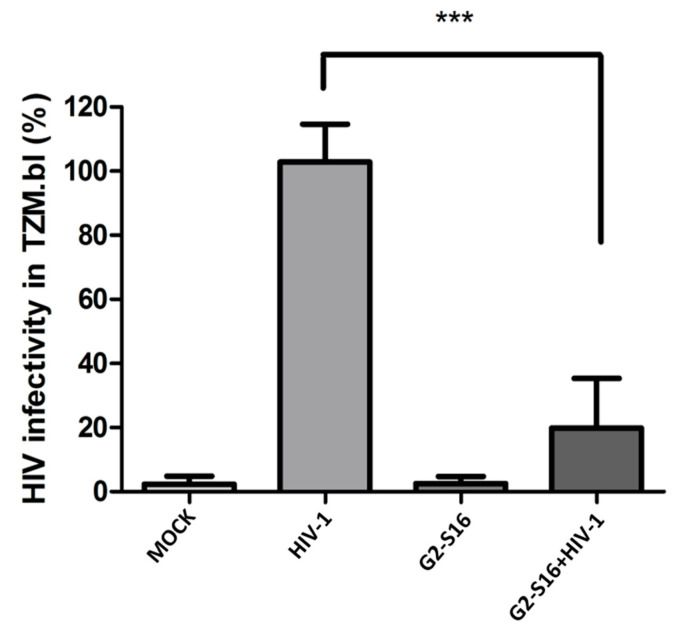
G2-S16 inhibits infection of T cells from infected MDMs. MDMs treated with G2-S16 and infected with 6 ng/µL of HIV-1 per 1 × 10^6^ cells for 2 h were co-cultured with isolated T cells for 48 h. After incubation, T CD4 cells were isolated and HIV-1 infectivity was titrated by infection of TZM.bl. Mock cells were used as the non-treated control. The mean values (mean ± SD) of three independent experiments are shown (*** *p* < 0.001).

In addition, in order to verify the infectivity of HIV-1 in these T cells, a titration in TZM.bl was performed (Figure 5). In this analysis, we observed that T lymphocytes treated with G2-S16 dendrimer carried just 20% of the infective viral particles that reached them from MDMs. Thus, this means that G2-S16 dendrimer is capable of not only minimizing the number of infectious viral particles that reach T CD4/CD8 cells, but also eliminating 80% of their infectivity in vitro.

## 3. Materials and Methods

### 3.1. Reagents

**Figure 6:**
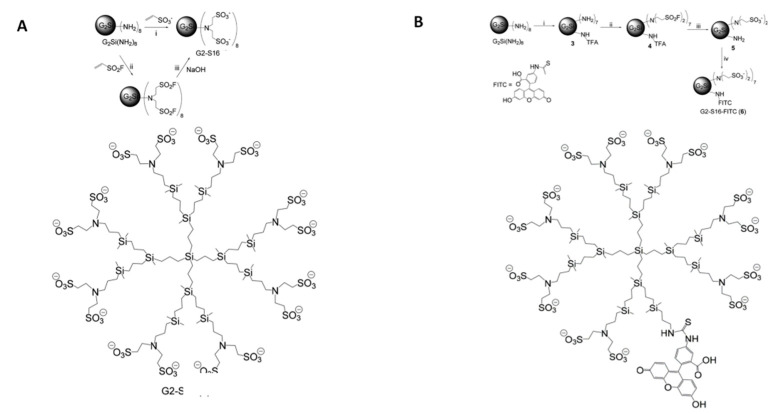
Schematic synthesis route and molecular representation of dendrimers. (**A**) G2-S16 with silicon core and 16 sulfonate end groups and (**B**) G2-S16-FITC with silicon core and 14 sulfonate end groups and FITC molecule were represented. The generation of dendrimers is determined by considering that each generation corresponds to the number of repeating layers of silicon atoms. Images adapted from Gutierrez-Ulloa et al. 2020.

G2-S16 and G2-S16-FITC anionic carbosilane dendrimers (Figure 6) with 16 functional groups sulfonates and silicon nucleus (C112H244N8Na16O48S16Si13; Mw = 3717.15 g/mol) were synthesized and analyzed according to the methods of the University Dendrimer Biomedical Applications group of University of Alcalá (UAH) [31] (Madrid, Spain). A 5 mM stock solution of G2-S16 dendrimer and subsequent dilutions to obtain µM concentrations were prepared in nuclease-free water (Promega, Madrid, Spain).

### 3.2. Cell Culture and Viral Strains

The TZM.bl cell line (ATCC, Manassas, NA, USA) derived from a human endometrial carcinoma was cultured in DMEM complemented with 5% FBS, 125 mg/mL ampicillin, 125 mg/mL cloxacillin, and 40 mg/mL gentamicin (Normon, Madrid, Spain).

Viral stocks of the CCR5-tropic R5-HIV-1NLAD8 laboratory strain were obtained by transient transfection of pNLAD8 plasmid (NIH AIDS Research and Reference Reagent Program [ARRRP]) in the HEK-293T cell line (ATCC, Manassas, VA, USA). Stocks were clarified before the evaluation of viral titter by HIVp24gag enzyme-linked immunosorbent assay (ELISA kit INNOTEST; Innogenetics, Ghent, Belgium).

### 3.3. Primary Cell Cultures, Purification, and Differentiation

Human peripheral blood mononuclear cells (PBMCs) were isolated from buffy coats obtained from anonymized healthy blood donors (Transfusion Centre of Madrid) following national guidelines. PBMCs were isolated by a standard Ficoll-Hypaque density gradient (Rafer, Madrid, Spain) and cultured following the procedures of Spanish HIV HGM BioBank [32]. Monocytes were purified using immune-magnetic antiCD14 microbeads (Miltenyi, Madrid, Spain), seeded at 1 × 10^6^ cells/mL, and cultured for 7 consecutive days in RPMI-1640 medium supplemented with 10% FBS, 1% l-glutamine, and 10 ng/mL of rhGM-CSF (Immunotools) to generate monocyte-derived macrophages (MDMs).

CD4/CD8 T cells were purified from PBMCs using immunomagnetic anti-Pan T microbeads (Pan T Cell Isolation Kit, human MicroBeads; Miltenyi Biotec, Bergisch Gladbach, Germany). Purified T cells were frozen at −80 °C for later use. T cells were seeded at 5 × 10^6^ cells/mL in RPMI 1640 medium (Biochrom AG, Berlin, Germany) with 10% fetal bovine serum (FBS; Gibco), 1% l-glutamine (Lonza, Walkersville, MD, USA), antibiotic cocktail (125 mg/mL ampicillin, 125 mg/mL cloxacillin, 40 mg/mL gentamicin (Normon, Madrid, Spain), and 60 U/mL of IL-2.

### 3.4. Immuno Fluorescence and Confocal Images

First, 0.5 × 10^6^ monocyte cells isolated and purified from PBMCs were incubated in 24-well plates for maturation to MDMs. After 7 consecutive days of incubation, MDMs were treated with G2-S16-FITC for 2 h at 37 °C. After incubation, medium was discarded, cells were rinsed with pH 3 glycine to detach non-internalized dendrimers, and fresh medium was added to cell cultures and incubated for 2, 4, 6, 24, 48, and 72 h. Then, supernatant was discarded, and cells were fixed in 4% paraformaldehyde (PFA; Panreac, Barcelona, Spain) for 10 min, washed 3 times in PBS, and permeabilized with 0.1% Triton X-100 (Sigma-Aldrich, St. Louis, MO, USA) for 10 min. After incubation, cells were washed 3 times in PBS, blocked with 1% bovine serum albumin (BSA Sigma-Aldrich, St. Louis, MO, USA) and 0.1%Triton X-100 in PBS for 30 min, and incubated with phalloidin ALEXA FLUOR^®^ 555 Conjugated antibody for 1 h. MDMs cells were washed 3 times in PBS and stained with 4′,6-diamidino-2- phenylindole (DAPI) (Sigma-Aldrich, St. Louis, MO, USA) for 10 min. Finally, cover glasses were mounted in slides and analyzed in a Leica TSC SPE confocal microscope (Leica Microsystems, Wetzlar, Germany). Fluorescence was analyzed using ImageJ software (National Institute of Health, Maryland, NA, USA).

### 3.5. Inhibition Assay

The potential activity of G2-S16 dendrimer against R5-HIV-1_NLAD8_ infection was measured by infectivity of HIV-1. For MDMs maturation, 1 × 10^6^ cells/well were seeded in 12-well plates and incubated at 37 °C for 7 days. After incubation, MDMs were treated with G2-S16 dendrimer for 1 h at 37 °C, washed with acid glycine, and infected with 6 ng/µL of R5-HIV-1_NLAD8_ for 2 h at 37 °C. Inoculum was discarded and RPMI-1640 medium supplemented with 10% FBS, 1% l-glutamine. The MDMs were incubated for 3 days. Then, the supernatants were collected, and cells were scraped in a solution of PBS (1% BSA, 2 mM EDTA) on ice. To quantify HIV-1 infectious viral particles, supernatants and pellets were analyzed by titration on TZM.bl cells.

### 3.6. Inhibition Assay Cell–Cell

After MDMs maturation, cells were treated with a mix 9/1 of G2-S16/G2-S16-FITC dendrimer for 2 h and infected with HIV-1 for 3 days. T CD4/CD8 cells isolated as described before were added to the culture at 1 × 10^6^ cells/mL and incubated for 48 h. After incubation, medium was collected in tubes and T CD4/CD8 cells were isolated by several washes with PBS and added to the tubes. Tubes were centrifuged at 1500 RPM for 10 min. Either cell pellets or supernatants were titrated for HIV-1 infectivity in the TZM.bl cell line.

### 3.7. Infectivity of HIV-1

The infectivity of the R5-HIV-1NLAD8 was measured by luciferase activity assay following the manufacturer’s protocol (Promega Corporation, WI, USA). Briefly, TZM.bl cells were seeded in 96-well plates at 1 × 10^4^ cell/well for 24 h. After incubation, medium was discarded and replaced with 100 μL of supernatants or cell pellets for 48 h. TZM.bl cells were lysed, and luciferase activity was measured at 570/650 nm in a BioTek Synergy™ 4 Hybrid Microplate Reader.

### 3.8. Determination of HIV-1 Quantity

In order to determine the R5-HIV-1_NLAD8_ quantity, each supernatant and cell pellet was analyzed by enzyme immunoassay for the quantitative detection of HIV-1 p24 antigen (INNOTEST HIV Antigen mAb, FUJIREBIO, Ghent, Belgium) following the manufacturer’s instructions. Supernatants and pellets were incubated in a 96-well plate coated with p24 antigen, and the absorbance was measured by the presence of viral protein HIV-1 p24 at 450 nm in a microplate reader BioTek Synergy™ 4 Hybrid Microplate Reader (BioTek, Winooski, NA, USA).

### 3.9. Statistical Analysis

Statistical analysis of the quantity and infectivity of HIV-1 was performed with GraphPad Prism v5.0 software (GraphPad, CA, USA) using the nonparametric unpaired *t*-test. Differences were considered significant at *p* < 0.05 (*), *p* < 0.01 (**), and *p* < 0.001 (***). All data were obtained from three independent experiments performed in triplicate.

## 4. Conclusions

In conclusion, our results indicates that G2-S16 dendrimer can act in different stages of HIV-1 infection. The main objective of G2-S16 dendrimer is its use as a microbicide, creating a physical barrier that protects the vaginal mucosa from being disrupted [33,34] and inhibiting several viral infections [35,36,37,38]. Thus, these results exacerbate the applicability of this G2-S16 dendrimer, as it is capable of inhibiting macrophages from spreading to other target cells, minimizing viral charge [39], as well as reducing viral reservoirs and sanctuaries due to the ability of macrophages to deliver viral particles to different anatomic targets. These results along with other results obtained by our group ease the way of G2-S16 to clinical trials.

## Data Availability

The data presented in this study are available on request from the corresponding author.
